# A multidisciplinary multicentric approach to the identification of predictive biomarkers of response to erenumab – the protocol of the phase 4, European, open-label controlled trial of the BIOMIGA project

**DOI:** 10.3389/fneur.2026.1878729

**Published:** 2026-08-31

**Authors:** Daniele Martinelli, Edoardo Caronna, Hauke Basedau, Rosaria Greco, Maria Magdalena Pocora, Max Schönthaler, Roberto De Icco, Gloria Vaghi, Grazia Sances, Natascia Ghiotto, Elena Guaschino, Marta Allena, Chiara Demartini, Miriam Francavilla, Anna Pichiecchio, Rut Mas-de-les-Valls, Deborah Pareto, Àlex Rovira, Victor J. Gallardo, Laila Asskour, Eulalia Gine-Cipres, Alicia Alpuente, Marta Torres-Ferrús, Elena Ballante, Gloria Castellazzi, Jan Mehnert, Arne May, Patricia Pozo-Rosich, Cristina Tassorelli

**Affiliations:** 1 Headache Science and Rehabilitation Unit, IRCCS C. Mondino Foundation, Pavia, Italy; 2 Headache and Neurological Pain Research Group, Vall d’Hebron Institute of Research (VHIR), Barcelona, Spain; 3 Headache Clinic, Neurology Department, Vall d’Hebron University Hospital, Barcelona, Spain; 4 Department of Systems Neuroscience, University Medical Center Hamburg-Eppendorf, Hamburg, Germany; 5 Brain and Behavioural Science Department, University of Pavia, Pavia, Italy; 6 Neuroradiology Unit, IRCCS C. Mondino Foundation, Pavia, Italy; 7 Section of Neuroradiology, Radiology Department, Hospital Universitari Vall d’Hebron, Barcelona, Spain; 8 Department of Political and Social Sciences, University of Pavia, Pavia, Italy

**Keywords:** biomarkers, CGRP - calcitonin gene-related peptide, machine learning, migraine, precision medicine

## Abstract

**Background:**

Migraine is a highly prevalent disease and the leading cause of years lived with disability in women. The development of therapies targeting the calcitonin gene-related peptide (CGRP) pathway has provided the first mechanism-based approach for migraine prevention. Although these treatments have demonstrated substantial efficacy, 30–50% of patients do not achieve an adequate clinical response. This variability underscores the need of prospectively identifying responders and non-responders, thereby enabling precise treatment allocation and improving clinical outcomes.

**Objectives:**

BIOMIGA is a multicentre phase IV study aimed at identifying biomarkers of response to anti-CGRP therapy in migraine through integrated clinical, molecular, genetic, and neuroimaging profiling, with patients stratified according to treatment response after 3 months treatment cycle with an mAB targeting the CGRP receptor (erenumab).

**Methods:**

Individuals with episodic and chronic migraine eligible for treatment with erenumab are enrolled along with healthy control subjects. Biomarkers are collected longitudinally at baseline and at the end of a 3-month treatment with erenumab. Circulating levels of CGRP and cytokines, genetic polymorphisms, and DNA methylation are assessed. Advanced structural and functional MRI scans are performed simultaneously, and comprehensive psychometric scales are used to map central pain processing and clinical burden. The final multimodal dataset is analysed using machine learning to develop and evaluate integrated models of treatment response.

**Expected results:**

The integration of multimodal data from patients and healthy controls, stratified according to treatment response, will enable the identification of a panel of biomarkers predictive of response to anti-CGRP therapy.

**Conclusion:**

The BIOMIGA trial represents a major step forward in the management of migraine. By combining a comprehensive multimodal profiling with a real-world, multicentre cohort of patients treated with erenumab, the study provides a framework for identifying robust biomarkers of treatment response and lays the foundation for truly individualised migraine prevention strategies.

**Clinical trial registration:**

ClinicalTrials.gov, identifier NCT04503083.

## Background

Migraine is a complex and highly prevalent neurological disorder (1) characterised by the abnormal processing of internal and external stimuli with recurrent activation of the trigeminovascular system. The release of vasoactive neuropeptides, particularly calcitonin gene-related peptide (CGRP), from trigeminal afferents in the meninges, promotes vasodilation, sterile neurogenic inflammation, and central sensitisation in brainstem and thalamic networks (2). These mechanistics insights have led to the development of preventive and acute treatments that selectively target the CGRP pathway, including monoclonal antibodies (mABs) against CGRP or its receptor and the small-molecule CGRP receptor antagonists (gepants).

CGRP-targeting mABs are large molecules with limited penetration across the blood–brain barrier, minimal pharmacokinetic interactions and long half-life. Because of these characteristics, CGRP-targeting mABs are administered parenterally monthly or quarterly and ensure high adherence to treatment randomized controlled trials (RCTs) long-term extension studies (OLEs) and real-world (RWE) cohorts confirm sustained reduction in monthly migraine days,(MMDs) disability, and acute medication use, with favourable tolerability profiles in both episodic (EM) and chronic migraine (CM), including patients who have previously failed multiple preventive treatments (3–6). In light of the robust and high-quality evidence supporting their efficacy and safety (4), mABs targeting CGRP or its receptor should be considered a first-line preventive treatment for migraine (7). This position has been endorsed by several international and national scientific societies. However, implementation remains limited in many settings due to heterogeneous reimbursement policies, despite pharmacoeconomic evaluations consistently indicating that CGRP-targeting mABs and, in certain contexts, gepants are cost-effective for CM, particularly when considering indirect costs and productivity gains (8–10). In this context, identifying predictors of differential treatment response is crucial to inform equitable and evidence-based reimbursement strategies, ensuring appropriate patient selection while aligning clinical practice with current scientific recommendations.

However, only a subset of patients achieve a ≥ 50% or ≥75% reduction in migraine days with CGRP-pathway-targeted therapies, highlighting the need for precision medicine approaches capable of matching individuals to the most effective therapy (11). Central to such approaches is the identification of robust biomarkers that can predict the likelihood of response. Despite decades of investigation, no biomarker has yet been validated for routine clinical use in migraine, either for diagnosis, disease staging, or prediction of treatment response (12, 13).

Multiple classes of candidate biomarkers have been explored. Circulating peptide markers, including CGRP and related neuropeptides, show increases during spontaneous and induced attacks and correlate with headache frequency and acute medication intake, but inter-individual variability, methodological differences and overlapping values with controls have limited their clinical utility (14, 15). Peripheral microRNAs show changes in chronic migraine and medication overuse and may normalise after detoxification or anti CGRP preventative therapy, but findings remain inconsistent and based on small samples (16, 17). Integrated molecular models combining miRNA profiles and genetic risk score have recently shown high discriminative accuracy, supporting multi-omic approaches, but still lack proper accessibility (18). Neuroimaging studies have shown structural and functional changes in pain-processing networks. Multimodal MRI can differentiate between migraine patients and controls, although there are currently no reliable imaging predictors for predicting response to CGRP-targeted treatments (13, 19, 20). Single-nucleotide polymorphisms (SNPs) modestly contribute to the risk of migraines, while changes in methylation at CGRP-related loci may affect migraine chronicity and the response to treatment (21–23). Genome-wide methylation studies further support distinct epigenetic signatures in migraine, but their relationship with pharmacological response is still largely unexplored.

This persistent gap in the identification of a single biomarker likely reflects the complex and dynamic nature of migraine biology – with distinct attack, pre−/post-dromal and interictal phases – the multiplicity of implicated mediators CGRP, PACAP38 and other neuropeptides, monoamines, inflammatory pathways, miRNAs, etc., and the involvement of both peripheral and central structures, together with marked interindividual variability of clinical expression (24). Methodological heterogeneity across studies – including differences in sampling paradigms, assay techniques, patient populations and outcome definitions – has further contributed to inconsistent findings and limited precision of biomarker estimates (15).

The advent of migraine-specific preventive therapies targeting the CGRP pathway offers an unprecedented opportunity to stratify patients according to response to a well-defined molecular mechanism and to link biomarkers to a precise pharmacological target (11). In parallel, advances in high-throughput and systems biology approaches allow the integration of multi-omic signatures with deep clinical phenotyping, neuroimaging and digital biomarkers, while sophisticated animal models continue to refine mechanistic understanding of CGRP-related neuroinflammation (24, 25). Recent work indicates that artificial intelligence and machine-learning methods can extract predictive patterns of response to anti-CGRP mABs from large, heterogeneous datasets, although current models remain preliminary, require external validation and are not yet applicable in clinical practice (26–29).

The biomarkers selected for the BIOMIGA study reflect a unified pathophysiological rationale, and each domain detects a complementary level of CGRP-mediated trigeminal-vascular activation. Peripheral neuroinflammatory signalling is associated with circulating levels of CGRP and cytokines, which reflect the degree of trigeminal activation at the time of treatment. Individual biological susceptibility to dysregulation of CGRP signalling is assessed through genetic variants and DNA methylation profiles at loci within the CGRP pathway. Central network reorganisation, a downstream consequence of prolonged trigeminal-vascular input, is quantified through resting-state functional connectivity. Together, these converging signals at all levels, rather than a single marker, are expected to predict response to erenumab. The resulting predictive models is expected to guide treatment decisions in patients with severe disability, such as those with CM, and provide essential evidence to healthcare systems and payers to support the rational and equitable allocation of advanced therapies.

We therefore hypothesise that the assessment of a wide panel of migraine-relevant features will yield a composite biomarker profile capable of predicting the response to treatment with anti-CGRP mABs in migraine prevention.

**Figure 1:**
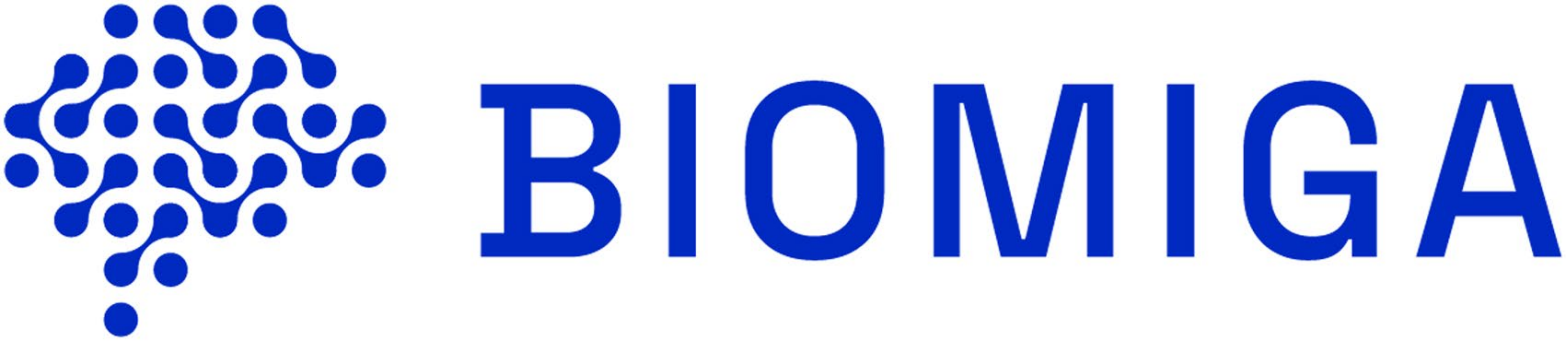
BIOMIGA logo: A multidisciplinary approach to the identification of biomarkers of migraine: A proof-of-concept study based on the stratification of responders to CGRP-targeting monoclonal antibodies.

To address this hypothesis, BIOMIGA (BIOmarkers of MIGraine) project (Figure 1) was designed as a proof-of-concept multidisciplinary study with the following specific objectives:

1. To define the clinical and omics differences between subjects with migraine stratified according to their response (responders vs. non-responders) to treatment with a CGRP-targeting mAb;
2. To identify a predictive profile using advanced machine learning techniques to analyze the integrated clinical and omics database generated from responder and non-responder data;
3. To integrate the findings from human studies with the evaluation of CGRP-targeting drugs in validated animal models of migraine.

In this manuscript, we will illustrate the BIOMIGA protocol that was devised to achieve objectives 1 and 2.

## Aim

Our primary working hypothesis is that patients with high-frequency episodic migraine (32) (HFEM) or CM who respond to treatment with a CGRP-targeting mAb (erenumab) exhibit a distinct clinico-omic profile compared with non-responders.

For this purpose, we will analyse a large set of variables collected from a cohort of subjects with HFEM or CM exposed to a 3-month cycle of migraine preventive treatment with a CGRP-targeting mAb. The large multimodal dataset will be analysed will be analysed with advanced multivariable modelling, including machine-learning and deep-learning techniques for feature selection, model building and internal validation. This process will identify distinct profiles associated with the responder or non-responder status, therefore providing the foreground to support personalised, cost-effective preventive treatment strategies in HFEM and CM.

## Methods and analysis

### Study design

The BIOMIGA trial is a multidisciplinary, multicentric, open-label, controlled, interventional, prospective phase 4 study aimed at identifying candidate biomarker profiles associated with response to CGRP-targeting mABs, using exploratory multivariable and machine-learning approaches to integrate demographic, clinical, psychological, cognitive, epigenetic, pharmacogenetic, biochemical, and structural and functional brain imaging data. The trial was registered at ClinicalTrials.gov with the ID NCT04503083.

### Sample size calculation

In the absence of a reliable biomarker for predicting the response to antimigraine drugs, we considered, on an empirical basis supported by the limited available data, the difference in baseline methylation levels of CpG islands of the RAMP1 gene, a key receptor subunit of calcitonin gene-related peptide (CGRP), between migraine subjects who are responders and those who are non-responders to CGRP-targeting mABs.

The minimum sample size was inferred considering previous exploratory epigenome-wide association scans (EWAS) in migraine. Wan D. et al. (16) found that the methylation level of CpG islands of the RAMP1 gene, a key receptor subunit of CGRP, was significantly lower in females living with migraine than in healthy female volunteers (2.18% ± 1.91% vs. 5.85% ± 5.41%). Taking this variance into account, we computed a sample size of 47 participants per group (80% power, 5% significance level). Moreover, we considered a 6% loss of participants. Given the rate of >75% response to erenumab observed in randomised clinical trials (30%), as well as the rate of <25% response, we required a minimum sample size of 168 subjects with migraine at baseline and 51 controls.

This sample-size calculation was designed to support the proof-of-concept biomarker-discovery component of the study and was not intended to provide statistical power for definitive high-dimensional machine-learning model development. Therefore, machine-learning analyses are considered exploratory and hypothesis-generating, with the aim of reducing dimensionality and prioritising candidate multimodal predictors for future validation in larger independent cohorts.

### Subjects recruitment

Participants were recruited in three tertiary headache centres: C. Mondino Foundation in Pavia (Italy), Vall d’Hebron University Hospital/Vall d’Hebron Institut de Recerca (VHIR) in Barcelona (Spain) and Institut für Systemische Neurowissenschaften, Universitätsklinikum Hamburg-Eppendorf Hamburg (Germany).

Each centre enrolled a group of patients with HFEM or CM and a group of age- and sex-matched healthy subjects.

### Eligibility criteria

#### Subjects living with migraine

##### Inclusion criteria

- Adults between 25 and 55 years of age of both sexes;
- European descent;
- Diagnosis of HFEM (patients fulfilling the diagnostic criteria for migraine with or without aura and experiencing 8–14 monthly headache days during the previous 3 months) or CM (≥15 headache days migraine/month, of which 8 have migraine characteristics for at least 12 months) according to the International Classification of Headache Disorders, 3rd edition (ICHD-3, 31).

##### Exclusion criteria

- a headache frequency of more than 25 days/month in the last 3 months;
- presence of acute medication overuse according to ICHD-3 criteria in the 3 months prior to the enrolment;
- any other significant medical condition (including neurological disorders, severe psychiatric illness, or cardiovascular disease);
- history or evidence of drug, or alcohol abuse or dependence within 12 months before the baseline visit;
- pregnant or breastfeeding women;
- women of childbearing potential (defined as all women physiologically capable of becoming pregnant) not using a reliable contraceptive method;
- concomitant use of other migraine preventive drugs.

#### Healthy controls

- Adults between 25 and 55 years of age of both sexes;
- European descent;
- Absence of any personal or first-degree familial history of recurrent primary or secondary headache disorders;
- Absence of any other significant medical condition (including neurological disorders, severe psychiatric illness, or cardiovascular disease);
- Absence of any history or evidence of drug, smoking, or alcohol abuse or dependence within 12 months before the baseline visit.

Pregnant or breastfeeding women were not included in this study.

### Interventions

#### Subjects living with HFEM or CM

They are assessed at baseline, then they receive a 3-month course of treatment with erenumab, a mAb targeting the CGRP receptor. The choice to use erenumab was dictated by the fact that it was the first and, at the time the trial began, the only CGRP-targeting mAb available on the European market.

No concomitant migraine preventive treatment was allowed during the screening, baseline and the 3-month treatment period. Abortive medications were allowed, but the type and formulation had to be the same throughout the study. Their usage was registered as well.

Their responders/non-responder status was defined assessed at the end of the 3-month treatment cycle, based on the headache diary data. Patients were classified as responders (R) if they achieved a ≥50% reduction in monthly headache days (MHD) during the final 4 weeks of the 12-week erenumab treatment period compared with the 4-week baseline run-in period. Patients who achieve a <50% reduction in MHD were classified as non-responders (NR). Within the R group, super responders (SR) were defined as patients who achieve a ≥ 75% reduction in MHD over the same period. Within the NR group, full non-responders (FNR) were defined as patients who achieve a < 25% reduction in MHD over the same period.

At visit 1, inclusion/exclusion criteria were reviewed; demographics and clinical characteristics were recorded. To avoid sample heterogeneity and to identify and minimise confounding factors, all participants underwent a structured interview to collect demographic information, including age, sex, ethnicity; clinical information including habits of caffeine, tobacco and drug consumption, physical activity level; life-time comorbidities, medication use, menstrual status and migraine-related information.

At this visit, participants started recording their headache characteristics in an *ad hoc* diary for a month to confirm the diagnosis at the following visit.

At visit 2, participants performed the following procedures:

- Diagnosis confirmation based on data recorded in the diary;
- Assessment of migraine-related disability, psychometric variables, quality of life, comorbidities, acute medications intake and Emergency Room Use in the previous month;
- Venous blood samples for DNA extraction for DNA methylation analysis for CGRP-related polymorphisms, and for biochemical profiling;
- Neuroimaging evaluation: 3 T Magnetic resonance imaging (MRI), including structural, resting state MRI (rsMRI), Arterial Spin Labelling (ASL) using a standardised protocol provided by UKE.

Once the assessment was completed, participants were administered the first 140-mg dose of erenumab. Upon discharge, the patient received 2 additional 140-mg doses of the drug, a training on the modality of self-injection and the schedule for self-injection (one dose every 28 days).

Visits 3 and 4 were remotely conducted to collect data from diaries, clinical scales and verify that the patients managed to self-inject the drug properly.

At visit 5, participants under:

- Assessment of treatment efficacy through the headache diaries and stratification of based on the response to treatment;
- Assessment of treatment satisfaction with validated patient-reported outcomes (PROs, listed in Table 1 and detailed in the next paragraph);
- Assessment of migraine-related disability, psychometric variables, quality of life, comorbidities, acute medications intake and Emergency Room use in the previous month; Assessment of epigenetic, biomolecular and neuroimaging parameters only for the SR and FNR subgroups.

**Table 1:** Scheduled activities for subjects with migraine and healthy controls.

	Subjects living with migraine					Healthy subjects
	Baseline (4 wk)	Treatment period				All activities were performed at visit 2
	Day 0	Day 1	Wk + 4	Wk +8	Wk +12 End of observ	
Visit number	1	2	3*	4*	5	
Informed consent	X					X
Pregnancy test	X	X				
Headache diary explained and given to patient	X	X	X	X	X	
Headache diary data collected		X	X	X	X	
Demographics, medical & headache history	X					X
Physical and neurological examination & vital signs	X				X	X
Adverse events		X	X	X	X	
Concomitant medications	X	X	X	X	X	
Blood samples§	X				X**	X
Neuroimaging	X				X**	X
Dosing		X	X	X		
MIDAS		X			X	
HIT-6		X			X	
BAI		X	X	X	X	X
BDI-II		X	X	X	X	X
MIG-SCOG		X			X	
MSQ		X			X	
PGI-I		X	X	X	X	

* Remote visit; ** Only for super responders and full non-responders. MIDAS, Migraine Disability Assessment; HIT-6, Headache Impact Test-6; BDI-II, Beck Depression Inventory-II; BAI, Beck Anxiety Inventory; MSQ, Migraine-Specific Quality of Life Questionnaire; MIG-SCOG, Migraine-Specific Cognitive Impairment Scale; PGI-I, Patient Global Impression of Improvement. § Pharmacogenetic polymorphisms, plasma levels of CGRP and cytokines.

#### Healthy controls

These subjects were only assessed at baseline (visit 2) to verify satisfaction of inclusion and exclusion criteria.

Once enrolled, blood samples were collected from the antecubital vein for DNA extraction for DNA methylation analysis for CGRP-related polymorphisms and for biochemical profiling. All subjects also underwent neuroimaging evaluation with 3 T Magnetic resonance imaging (MRI), including structural, resting state MRI (rsMRI), and Arterial Spin Labelling (ASL) using a standardised protocol provided by UKE.

**Figure 2:**
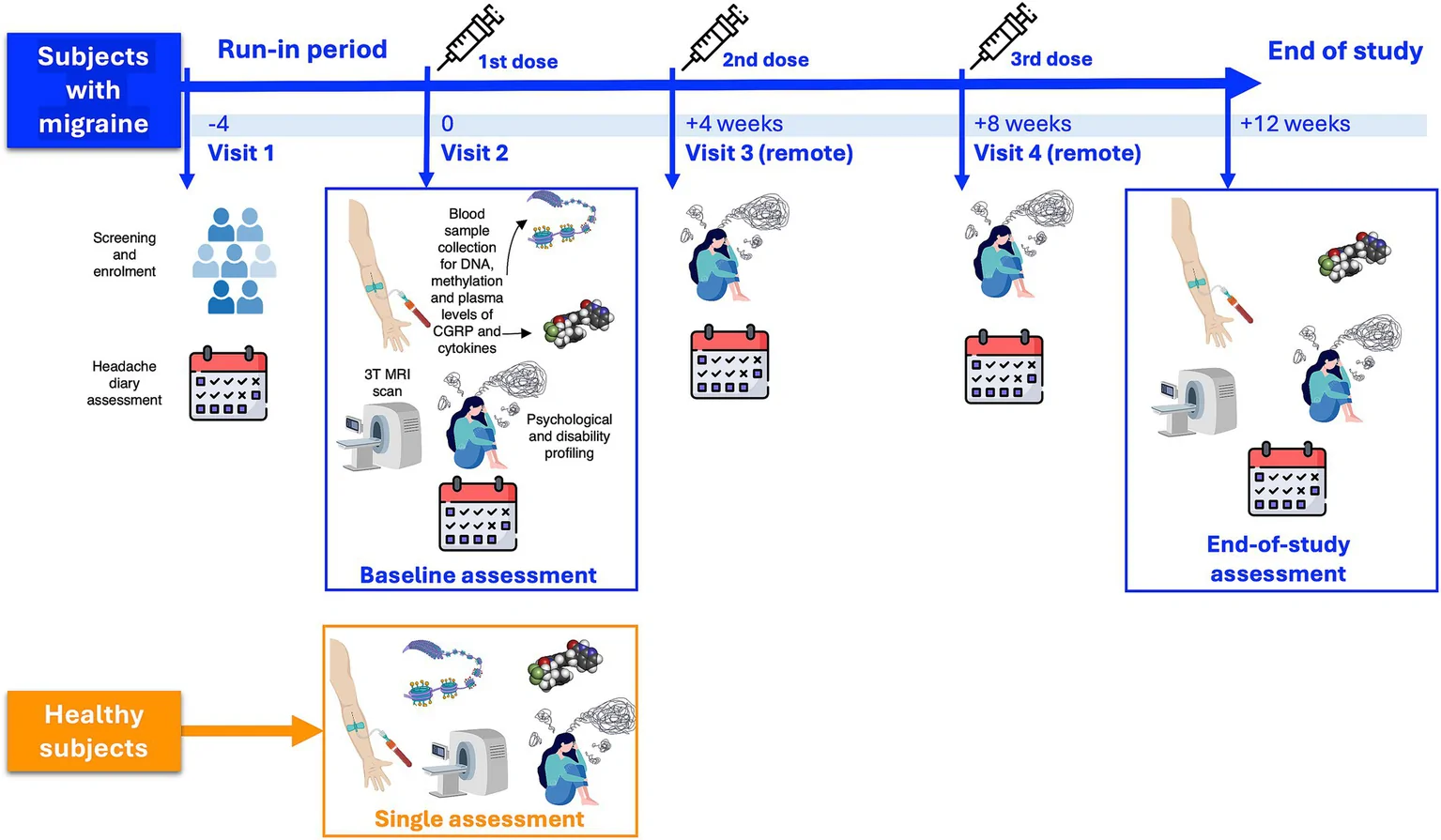
Graphical representation of the BIOMIGA trial and associated procedures. The study flow differentiates procedures for people living with migraine (blue line) and healthy controls (orange line).

The protocol design is illustrated in Figure 2, scheduled activities for subjects with migraine and healthy controls are summarised in Table 1.

### Data collection and management

#### Clinical profiling

The screening/baseline visit comprised the assessment of study eligibility, including a complete physical and neurological examination. A semi-structured interview was used to collect sociodemographic data, medical and headache history, detailed clinical characteristics, and current and past medication use.

Participants were instructed to fill out a prospective headache diary in paper format with daily entries throughout the baseline period and treatment period. Information about the abortive medication use was also acquired.

Participants were instructed to complete seven patient-reported outcome measures at screening, on Day 1 (first dose), and at the end of the treatment period. All questionnaires were administered in paper or digital format.

At baseline, disability was assessed with validated questionnaires in the national language of the participating centres. The questionnaires included the Migraine Disability Assessment (MIDAS) (30) and the Headache Impact Test-6 (HIT-6) (31); depression and anxiety symptoms with the Beck Depression Inventory-II (BDI-II) (32) and the Beck Anxiety Inventory (BAI) (33); health-related quality of life with the Migraine-Specific Quality of Life Questionnaire (MSQ) (34); and subjective cognitive impairment during migraine attacks with the Migraine-Specific Cognitive Impairment Scale (MIG-SCOG) (30). Information on medical comorbidities was also collected.

At all study visits, we recorded the use of concomitant medications and any adverse events (AEs) or serious adverse events (SAEs), which were graded according to the Common Terminology Criteria for Adverse Events (CTCAE) version 6.0 (35).

#### Biomolecular profiling

Blood samples (3 mL) were obtained from the cubital vein of participants, between 8:00 a.m. and 12:00 a.m. Patients were assessed during an interictal period and at least 12 h from the intake of an acute drug for migraine treatment. Venous blood was collected into one 9-mL K₃ ethylenediaminetetraacetic acid tube for plasma and two 8-mL sodium heparin Cell Preparation Tubes for mononuclear cell isolation.

For plasma levels of CGRP and cytokines, the K₃ tube with ethylenediaminetetraacetic acid (EDTA) was processed immediately after collection and centrifuged at 1500 × g for 15 min at room temperature; the plasma was separated.

For the expression of cytokine and CGRP mRNA, the cell preparation tubes were processed within 2 h of collection. The samples were centrifuged at 1,500–1,800 × g for 30 min at room temperature. Following centrifugation, the plasma was removed, and the mononuclear cell layer was transferred to 15 mL conical tubes that were free of DNase and RNase.

Fifteen milliliters of phosphate-buffered saline were added to the tube, gently mixed by inversion, and centrifuged again at 1,500 × g for 10 min. The supernatants were discarded, leaving the cell pellets for subsequent extraction.

Plasma aliquots were transferred into 1.5-mL polypropylene microtubes and stored at −80 °C for 4 months before assay. Mononuclear cell pellets from both conical tubes were pooled into a single 1.5-mL ribonuclease/deoxyribonuclease-free microtube, resuspended in 400 μL of Trizol reagent and stored at −80 °C until nucleic acid extraction. For centralised biomolecular analyses, both plasma samples and cell pellets in Trizol were shipped to the Pavia laboratory in temperature-controlled containers (dry-ice or liquid-nitrogen dry shippers), ensuring that a temperature of ≤ 80 °C was maintained throughout transport.

Pre-planned biomolecular analyses were performed at IRCCS Mondino laboratory, and they comprised the measurement of plasma α-CGRP, IL-1β, and TNF-α levels using a commercial enzyme-linked immunosorbent assay kit (α-CGRP, using OKEH00875 assay, Aviva Systems Biology; TNF-α, using RAB0476 assay and IL-1β using RAB0306 assay, by Millipore Sigma). Two measurements were performed for each sample, and the mean value was calculated and considered for statistical analysis.

In parallel, miRNA expression of CGRP, IL-1β and TNF-α was quantified in peripheral blood mononuclear cells by real-time quantitative polymerase chain reaction. The same peripheral profile of CGRP and pro-inflammatory cytokines was assessed at baseline in a group of healthy control subjects.

#### Pharmacogenetic profiling

For genomic analyses of DNA polymorphisms, venous blood was drawn by antecubital phlebotomy into a single 3-mL K₃-EDTA tube and stored at 4 °C for a maximum of 2 months before processing.

Samples were shipped to the central laboratory in Pavia in temperature-controlled conditions (chilled transport at 2–8 °C) for subsequent DNA extraction and genotyping.

Real-time polymerase chain reaction was used to identify single-nucleotide polymorphism variants of the calcitonin receptor-like receptor and some genes that modify receptor activity in all enrolled patients and healthy control subjects (36). Specifically, we selected single-nucleotide polymorphisms within RAMP1 (receptor activity–modifying protein 1) (37), CALCRL (calcitonin receptor-like receptor) and CRCP (CGRP receptor component protein), which together form and regulate the canonical calcitonin gene-related peptide receptor complex, as well as variants in GNAS, encoding the stimulatory alpha subunit of the heterotrimeric G protein that couples this receptor to intracellular signalling. These loci were chosen because prior evidence implicates them in migraine susceptibility and in the modulation of calcitonin gene-related peptide–mediated pathways, making them plausible candidates for influencing clinical response to calcitonin gene-related peptide–targeted preventive therapies (38).

#### Epigenetic profiling

For DNA methylation analyses, venous blood was collected into one 8-mL sodium heparin Cell Preparation Tube for mononuclear cell isolation. Samples were processed within 2 h of collection. Tubes were centrifuged at 1,500–1,800 × g for 30 min at room temperature, the plasma phase was removed, and the mononuclear cell layer was carefully transferred to a 15-mL deoxyribonuclease/ribonuclease-free conical tube. Phosphate-buffered saline was added to a final volume of 15 mL, the suspension was gently mixed by inversion and centrifuged at 1,500 × g for 10 min at room temperature. The supernatant was aspirated without disturbing the cell pellet.

The resulting mononuclear cell pellet in the deoxyribonuclease/ribonuclease-free conical tube was stored at −80 °C for a maximum of 4 months before DNA extraction. For centralised epigenetic analyses, frozen pellets were shipped to the Barcelona laboratory in temperature-controlled containers (liquid-nitrogen dry shipper or dry-ice shipper), ensuring maintenance of a temperature of ≤ 80 °C throughout transport.

Planned DNA methylation of CpG islands was quantified using the Infinium MethylationEPIC BeadChip (Illumina, Inc.) at Vall d’Hebron laboratories. All procedures were performed following the manufacturer’s recommended protocols. Briefly, 600 ng of total DNA was subjected to bisulfite conversion using the Zymo EZ DNA Methylation Kit. Processing of the EPIC v2 BeadChips was carried out using the automated Infinium HD Assay Methylation protocol (User Guide 15,019,519 v01) on a TECAN Freedom EVO robotic platform. BeadChips were scanned using the Illumina iScan system with Autoloader to generate .idat files, and data quality control was performed using GenomeStudio software (version 2011.1). Post-processing and downstream analyses are going to be reported in future publications.

#### Neuroimaging profiling

Magnetic resonance imaging was performed under standardised interictal conditions to minimise intra- and inter-individual variability with 3 tesla scanners. All participants had fasted for at least 3 h before the scan and refrained from coffee, tea and other stimulant drinks during the same interval. Scans were acquired when participants had been pain-free for at least 24 h and had not taken any acute migraine medication or benzodiazepines in the preceding 24 h; if a migraine attack occurred, imaging was rescheduled to a pain-free day. To further reduce intra-subject variability, each participant was scanned at approximately the same time of day at all visits.

Patient comfort and immobility were actively promoted. During resting-state functional imaging, the scanner-room lights were switched off, and participants were instructed to keep their eyes closed, remain awake and avoid focusing on any specific thought. The imaging protocol was acquired in a fixed order: high-resolution structural imaging (magnetisation-prepared rapid gradient echo), resting-state functional magnetic resonance imaging, and arterial spin labelling. The imaging protocol was set and harmonised between centres. When feasible, respiratory and cardiac signals (breathing, pulse and/or electrocardiogram) were recorded to allow correction of physiological artefacts in the resting-state functional data.

##### Acquisition protocol

Magnetic resonance imaging was performed at all participating centres using 3-Tesla MRI scanners, following a shared, harmonised acquisition framework specifically developed for the BIOMIGA project. Across the three sites (Pavia, Hamburg and Barcelona), the MRI protocol systematically included the same core set of sequences: (1) High-resolution three-dimensional T1-weighted structural imaging (3D T1); (2) Resting-state functional MRI (rs-fMRI); (3) Arterial spin labelling (ASL) perfusion imaging. Before proceeding with the analysis, all images were defaced locally to anonymise the dataset.

These sequences were selected to enable integrated assessment of brain morphology, functional connectivity and cerebral perfusion, which are all implicated in migraine pathophysiology and CGRP-related mechanisms.

At the coordinating centre in Pavia, MRI data were acquired on a 3.0 T Siemens Skyra scanner (Siemens Healthineers, Erlangen, Germany) equipped with a 32-channel head coil. The acquisition protocol comprised: (1) a high-resolution 3D T1-weighted scan (MPRAGE; TR/TE = 2300/2.98 ms, flip angle = 9°, 240 slices, FOV = 256 mm, voxel size = 1.0 mm^3^ isotropic) for anatomical reference and morphometric analyses; (2) resting-state fMRI (rs-fMRI) using a T2*-weighted gradient-echo echo-planar imaging (GRE-EPI) sequence (TR/TE = 2330/26 ms, voxel size = 2.0 mm^3^ isotropic, FOV = 256 mm, 34 slices, 305 volumes); (3) arterial spin labeling (ASL) using a 3D Turbo Gradient Spin Echo (TSGE) pseudo-continuous ASL (pCASL) sequence (TR/TE = 3000/22.56 ms, inversion time (TI) = 1800 ms, FOV = 256 mm, 42 slices, 2 measurements [1 label/ 1 control]).

At the coordinating centre in Barcelona, MRI data were acquired on two 3.0 T Siemens systems (Tim Trio and MAGNETOM Prisma, Siemens, Erlangen, Germany). The Tim Trio was equipped with a 32-channel phased-array head coil, whereas the MAGNETOM Prisma used a 64-channel head–neck coil. The acquisition protocol comprised: (1) a high-resolution 3D T1-weighted scan (MPRAGE; TR/TE = 2300/2.98 ms, FOV = 256 mm, 192 slices, voxel size = 1 mm^3^ isotropic); (2) resting-state fMRI (rs-fMRI) using a T2*-weighted GRE-EPI sequence (TR/TE = 2330/26 ms, voxel size = 2.0 mm^3^ isotropic, FOV = 224 mm, 34 slices, 305 volumes); (3) arterial spin labeling (ASL) using a 3D TSGE pCASL sequence (TR/TE = 3000/22.56 ms, TI = 1800 ms, FOV = 256 mm, 42 slices, 2 measurements [1 label/1 control], voxel size = 1.0 × 1.0 × 2.5 mm^3^).

In Hamburg, MRI data were acquired on a 3.0 T MAGNETOM Prisma scanner (Siemens Healthineers, Erlangen, Germany) equipped with a 32-channel head coil. The acquisition protocol comprised: (1) a high-resolution 3D T1-weighted scan (MPRAGE; TR/TE = 2300/2.98 ms, flip angle = 9°, 240 slices, voxel size = 1 mm^3^ isotropic, FOV = 256 mm); (2) resting-state fMRI (rs-fMRI) using a T2*-weighted GRE-EPI sequence (TR/TE = 2330/26 ms, voxel size = 2 mm^3^ isotropic, FOV = 256 mm, 34 slices, 305 volumes); and (3) arterial spin labeling (ASL), including a 3D TSGE pCASL sequence (TR/TE = 3000/22.56 ms, TI = 1800 ms, FOV = 256 mm, 42 slices, 2 measurements [label/control]) and an additional 2D pulsed ASL (PASL) sequence (TR/TE = 3000/12.0 ms, TI = 1800 ms, FOV = 256 mm, 21 slices, 90 measurements [45 label/45 control]).

To account for multicentric variability, scanner site was explicitly modelled as a covariate in all group-level neuroimaging analyses, and dedicated quality-control procedures were applied to ensure consistency of spatial coverage, artefact burden and temporal signal characteristics across centres. All datasets underwent local anonymisation and active defacing before central storage and processing.

Details of pre- and post-processing are going to be integrated in future publications.

Additionally, BOLD activation imaging using an event-related fMRI pain paradigm were carried out in an independent subset of 25 CM patients, before and after treatment, to investigate treatment-related modulation of trigeminovascular and brainstem pain-processing circuits. To this end, we used an adapted imaging protocol with a nociceptive stimulation paradigm designed to engage the trigemino-thalamic and spino-thalamic pathways, with a particular focus on brainstem nuclei involved in migraine pathophysiology, including the spinal trigeminal nucleus, periaqueductal grey, locus coeruleus and dorsal raphe complex. This paradigm was adapted from previously validated high-resolution brainstem fMRI protocols and optimised to detect subtle changes in nociceptive processing and descending pain modulation. The experimental design allowed investigation of afferent sensory processing as well as efferent modulatory mechanisms, testing the hypothesis that peripheral blockade of the CGRP receptor by erenumab induces measurable central changes in pain-related brainstem and thalamo-cortical networks.

To complement central neuroimaging findings with a peripheral functional readout, the same subgroup underwent assessment of neurovascular skin responses to controlled thermal stimulation. A moderate heat stimulus (40 °C) was applied to the left volar forearm, and the resulting axon-reflex-mediated vasodilatory flare was quantified using laser speckle contrast imaging (PeriCam PSI System®, Perimed Instruments, Sweden). This approach provides a non-invasive measure of peripheral CGRP-dependent neurovascular reactivity. By investigating both task-based fMRI of the brain and peripheral neurovascular measurements, this experimental model aimed at bridging molecular blockade at the periphery with functional reorganisation of central pain networks.

### Statistical analysis

#### Efficacy endpoint analyses

The primary objective of the study is to identify a panel of biomarkers that is reliably associated with response to treatment in a substantial proportion of patients with migraine. To this end, we will develop classification algorithms that assign patients to response categories defined on an ordinal scale. Supervised machine-learning approaches based on parametric, semi-parametric and non-parametric models are going to be fitted and compared. The optimal model will be selected according to predefined performance criteria, including area under the curve (AUC), accuracy, precision and recall.

Secondary analyses will characterise differences between migraine patients and healthy controls at baseline in: (i) DNA methylation profiles; (ii) structural and functional magnetic resonance imaging measures; and (iii) pharmacogenetic, biochemical, clinical and psychological variables. Within the migraine cohort, we will also compare DNA methylation, brain morphometry and functional connectivity, as well as pharmacogenetic, biochemical, clinical and psychological measures, among patients classified as responders and non-responders to CGRP-targeting mABs.

Given the prospective design, changes in clinical status, disability and psychometric measures, from baseline to the end of the treatment period, are going to be evaluated to quantify the impact of the therapy and to relate these changes to treatment response categories. In parallel, longitudinal differences in structural and functional magnetic resonance imaging measures among response groups are going to be examined to identify treatment-related brain changes and estimate their effect sizes in relation to drug exposure and clinical improvement (e.g., reduction in monthly headache days).

Finally, plasma concentrations of CGRP, IL-1β and TNF-*α*, together with their messenger RNA expression in peripheral blood mononuclear cells, are going to be assessed over the course of treatment to explore treatment-related alterations in peripheral inflammation and their correlation with clinical endpoints, including headache frequency, acute medication intake and disability.

#### Handling missing data

Missing values in clinical and psychometric variables (BAI, BDI-II, MIDAS, HIT-6, MSQ total score), as well as missing body mass index (BMI) values, were handled using multiple imputation by chained equations (MICE) implemented in R (2), based on Fully Conditional Specification, whereby each incomplete variable was imputed using a separate regression model appropriate to its distribution. Regarding neuroimaging data, missing or incomplete MRI datasets were not imputed. Across centres, minor differences in acquisition resulted in variable numbers of volumes, particularly in resting-state fMRI sequences. To ensure comparability across sites, resting-state datasets were temporally harmonised by retaining an equivalent number of volumes for all subjects included in the analyses.

In addition, datasets affected by excessive head motion, defined as motion artefacts that could not be adequately corrected using standard motion-correction algorithms during preprocessing, were excluded from neuroimaging analyses. This quality-control step was applied uniformly across centres to minimise motion-related bias and to preserve the validity of functional connectivity and perfusion measures.

No imputation was performed for missing neuroimaging data; analyses were conducted on quality-controlled datasets only.

#### Data quality assessment

Each participating centre was responsible for the collection, entry and local storage of clinical and biological data, and for implementing site-specific monitoring procedures in accordance with national regulations for open-label phase IV studies. Cross-centre checks of data quality and internal consistency were periodically performed, and any discrepancies were resolved through review of source documents and discussion between investigators until consensus was reached (39).

#### Descriptive and inferential statistics

All computations were conducted via *R* software (version 4.4.0). Quantitative variables were compared using the cross-sectional Mann–Whitney test, whereas categorical variables were assessed via the chi-square test or Fisher’s exact test. A value of *p* < 0.05 was considered statistically significant. The statistical plan included baseline comparisons between R and NR across clinical, anamnestic, psychological, and disability measures. Variables were also compared between healthy controls and migraine patients, stratified for gender and diagnosis. Predictive thresholds for circulating serum *α*-CGRP, IL-1β, and TNF-α levels were estimated in patients using binomial logistic regression (generalised linear model, GLM).

#### Mitigation of confounding factors

Given the real-world observational design of the BIOMIGA study, several measures were adopted to minimise the influence of potential confounding factors. Clinical outcome classification were based on prospectively collected headache diaries, which represent the reference standard for the assessment of migraine outcomes in real-world studies and reduce recall bias through systematic longitudinal recording of headache frequency and acute medication use (39). Standardised eligibility criteria limited major sources of confounding, including medication overuse headache, concomitant preventive treatments, severe neurological or psychiatric disorders, cardiovascular diseases, substance abuse, and other medical conditions potentially affecting biomarker levels or treatment response.

Demographic and clinical variables, including age, sex, migraine subtype, disease duration, baseline monthly migraine days, disability measures, acute medication intake, and relevant comorbidities, were systematically collected and evaluated as potential confounders. Analyses aimed at identifying clinically meaningful cut-offs for circulating CGRP concentrations were performed using GLM for prespecified clinical and demographic covariates to estimate independent associations while accounting for potential confounding. For epigenetic analyses, standardised procedures were adopted to minimise pre-analytical variability. Quality-control procedures and batch-effect correction methods were applied where appropriate, and known determinants of DNA methylation variability, including age, sex, medication exposure, and lifestyle-related factors such as smoking habits, were considered during statistical analyses to reduce potential confounding.

To reduce variability in neuroimaging-derived measures, MRI data were acquired using standardised acquisition protocols and processed through harmonised quality-control and preprocessing pipelines. Structural and functional MRI datasets underwent visual and automated quality assessment, with exclusion of scans affected by major artefacts or excessive motion.

Established preprocessing procedures, including motion correction, spatial normalisation, and nuisance signal regression for functional MRI data, were applied to minimise non-biological sources of variability. In addition, demographic and imaging-related factors known to influence brain measures, such as age, sex, and head motion parameters, are going to be considered during statistical modelling and feature selection procedures.

#### Machine learning

##### Preprocessing and feature selection

Given the multimodal nature of the BIOMIGA dataset and the high dimensionality arising from the integration of clinical, psychometric, biomolecular, genetic, epigenetic and neuroimaging variables, a structured preprocessing and feature-selection pipeline are going to be implemented to reduce dimensionality, limit multicollinearity and minimise the risk of overfitting.

Continuous variables are going to be inspected for distributional properties and standardised when appropriate. Categorical variables are going to be encoded using dummy variables. Pairwise correlation analysis is going to be performed to identify highly collinear features, and redundant variables will be excluded before model training.

An initial univariate feature-screening step is going to be applied to reduce the feature space. Candidate predictors is going to be tested for their association with the target variable (treatment response category) using appropriate statistical tests (*t*-test or ANOVA for continuous variables; *χ*^2^ test for categorical variables). Variables not showing evidence of association is going to be excluded from subsequent modelling. This step is going to be used exclusively for dimensionality reduction and not for inferential purposes.

To prevent information leakage, all preprocessing and feature-selection steps is going to be performed within the training data only, following best practices for predictive modelling. Given the protocol-oriented nature of the present manuscript and the proof-of-concept design of BIOMIGA, the machine-learning analyses are planned as an exploratory predictive modelling component intended to generate and prioritise candidate multimodal predictors of treatment response, rather than to provide a definitive externally validated clinical prediction tool.

##### Handling of class imbalance

Given the expected imbalance between response categories (e.g., super-responders, responders, partial responders and non-responders), class imbalance is going to be explicitly addressed during model training. For Random Forest (RF) models, class imbalance is going to be handled by applying class-weighted training, assigning higher misclassification penalties to underrepresented classes. For algorithms requiring explicit balancing, resampling strategies within the training folds is going to be considered while preserving the original class distribution in the test data. Model performance is therefore going to be evaluated using metrics that are robust to class imbalance, rather than relying solely on overall accuracy.

##### Machine learning (ML) algorithms

As the primary modelling approach, RF classification was selected because it is well suited to high-dimensional biomedical datasets, can model non-linear relationships and interactions among predictors, and provides internal estimates of generalisation error through out-of-bag (OOB) samples. RF models is going to be trained using bootstrap aggregation, with random feature selection at each node split. Hyperparameter tuning will consider the number of trees, the number of variables sampled at each split, tree depth and terminal node size; final values are going to be selected according to cross-validated/OOB performance. RF-derived feature-importance measures are going to be used to rank predictors according to their contribution to classification performance. This approach is consistent with recent migraine studies using ML to predict response to anti-CGRP monoclonal antibodies (26, 28).

To assess the incremental predictive value of multimodal data integration, RF models are going to be developed using a progressive feature expansion strategy. Models are going to be trained and compared using: (i) clinical and biochemical variables; (ii) clinical, biochemical, and genetic variables; (iii) clinical, biochemical, genetic, and epigenetic variables; (iv) clinical, biochemical, genetic, epigenetic, and structural MRI variables; and (v) the full multimodal dataset including clinical, biochemical, genetic, epigenetic, structural MRI, and functional MRI (fMRI) features. This sequential approach will quantify the added predictive contribution of each data modality and reduce the likelihood that model performance is driven by a single data source or modality-specific confounding effect.

Following this initial phase, a stricter feature-selection step based on RF importance rankings is going to be applied to identify a reduced and more informative feature subset. This reduced feature space will then be used to train and compare additional supervised machine-learning algorithms, including:

- Support vector machine (SVM): Linear and radial-basis-function kernels are going to be considered to test whether response categories can be separated by maximum-margin decision boundaries in the selected feature space. The regularisation parameter and, for radial-basis-function kernels, the gamma parameter is going to be tuned within the inner validation loop. SVMs have previously been applied to migraine classification using clinical and neuroimaging-derived variables (40).
- Artificial neural network (ANN): simple feed-forward multilayer perceptron models are going to be considered to capture potential non-linear relationships among multimodal predictors. Given the limited sample size, the ANN architecture is going to be intentionally parsimonious, with tuning of the number of hidden units/layers, regularisation strength and learning rate, and with early stopping where appropriate to reduce overfitting. Neural-network approaches are included as exploratory non-linear benchmark models in line with the broader use of artificial intelligence methods in migraine research (29).
- k-Nearest Neighbour (kNN): Distance-based classification is going to be applied after appropriate scaling of continuous predictors. The number of neighbours and distance metric are going to be selected within the training folds. kNN will serve as a simple, non-parametric benchmark model that classifies subjects according to similarity with neighbouring cases in the reduced multidimensional biomarker space (41)
- Decision Tree: classification-tree models are going to be used as interpretable non-linear classifiers able to represent threshold-based combinations of predictors. Tree depth, minimum node size and pruning/complexity parameters are going to be tuned to limit overfitting. Decision-tree models will also provide a clinically intuitive comparator to ensemble methods such as RF and AdaBoost (40)
- AdaBoost: adaptive boosting models based on weak decision-tree learners are going to be considered to assess whether sequential reweighting of misclassified observations improves classification. The number of estimators, learning rate and base-tree complexity are going to be are going to be tuned within the inner validation loop. Boosting approaches, including AdaBoost, have previously been used in migraine classification studies combining questionnaire and imaging-derived variables (40, 42, 43, 54).

#### Model evaluation and validation

These models are going to be evaluated within a nested cross-validation framework, with inner loops for hyperparameter tuning and outer loops for performance estimation, to ensure an unbiased assessment of generalisation performance. All transformations, feature-selection procedures, class-balancing strategies and hyperparameter optimisation steps are going to be confined to the training folds and then applied to the corresponding validation/test folds. For RF models, predictive performance will also be assessed using the out-of-bag (OOB) error rate, which provides an internal estimate of generalisation performance without the need for a separate validation set. OOB misclassification rates and class-specific OOB errors are going to be examined as global and category-specific performance indicators. For models evaluated using cross-validation, performance is going to be quantified using a combination of metrics, including balanced accuracy, sensitivity, specificity, positive and negative predictive values and area under the receiver operating characteristic curve (AUC), allowing comparison across algorithms in the presence of class imbalance.

**Figure 3:**
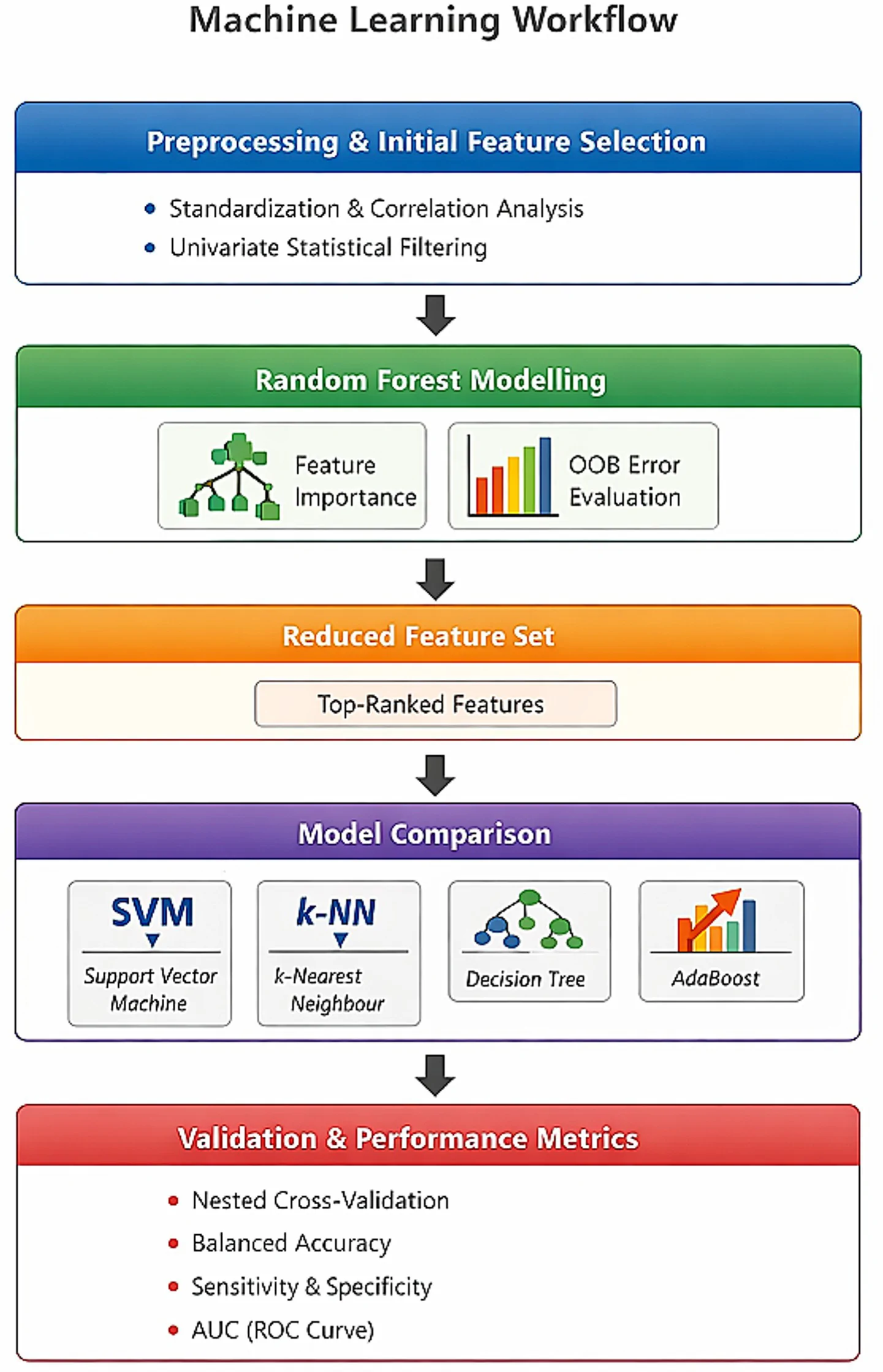
Schematic representation of the machine-learning pipeline applied to the BIOMIGA multimodal dataset.

This multi-step modelling strategy was designed to balance predictive performance, robustness and interpretability in the context of a complex, high-dimensional biomarker dataset. Because the final model specification depends on the observed data structure and on the retained feature set, the complete algorithmic configuration, selected hyperparameters and final validation results are going to be reported in the subsequent results manuscript. A schematic overview of the machine-learning pipeline, including preprocessing, feature selection, model training, validation and performance evaluation, is provided in Figure 3. This workflow illustrates the sequential and nested structure of the analysis and highlights the measures adopted to minimise overfitting and information leakage.

## Status of the study

The enrolment of participants has been concluded in January 2024. Data cleaning and anonymisation/pseudonymization were completed in July 2024. Data processing for baseline values was completed in May 2025, and baseline data are undergoing the last round of revision from the authors.

Data processing for the analysis of stratified groups is presently ongoing.

## Legal aspects, ethics and dissemination

### BIOMIGA consortium

The BIOMIGA consortium was established within the ERA-Net NEURON programme (Network of European Funding for Neuroscience Research), Joint Transnational Call 2019 “European Research Projects on Biomarkers,” and brings together three complementary centres of excellence in headache research and care: IRCCS Mondino Foundation (Pavia, Italy; coordinating centre), Vall d’Hebron University Hospital (Barcelona, Spain) and the University Medical Centre Hamburg-Eppendorf (Hamburg, Germany). Fundamental research activities commenced on 25 April 2020 and clinical recruitment on 16 June 2020. Recruitment was completed on the 30th of October 2023 and data collection was completed by January 2024. The project ended on 11 September 2024.

A key strength of the consortium is the implementation of harmonised procedures for clinical, radiological and biological data collection across all sites, achieved through a shared, detailed protocol and standard operating procedures, complemented by a training video to ensure correct handling and processing of peripheral blood mononuclear cells.

Overall coordination and scientific leadership were provided by the principal investigator, Cristina Tassorelli (IRCCS Mondino Foundation), and local principal investigators at each centre: Patricia Pozo-Rosich at Vall d’Hebron University Hospital and Arne May at Hamburg University.

### Ethics approval and consent

This study was conducted in accordance with the International Conference on Harmonisation note for guidance on Good Clinical Practice. The Biomiga Protocol received the following approval from each local ethical committee:

1. Pavia Ethical Committee, San Matteo Hospital, n. 20200048923;
2. Ethics committee of the Ärztekammer Hamburg, PV7356;
3. Vall d’Hebron Ethics Committee, AC19/00214.

The study was originally planned to be completed within 3 years; however, due to the COVID-19 pandemic and its impact on clinical and research activities, including restricted access to hospitals and reduced enrolment rates, a 1-year extension was granted by the funding body and duly notified to all relevant Ethics Committees, with no further amendments to the study protocol required.

All participants were fully informed about the study, including the risks and benefits of their participation in the study. Any participant could withdraw from the study at any time, for any reason, specified or unspecified, and without penalty or loss of benefits to which the patient is otherwise entitled. No study-related procedures, including any screening procedures, were performed before the investigator had obtained written informed consent from the participants.

All the information obtained in the study are treated confidentially, complying with the GDPR 2016/679 and the Organic Law of Protection of Personal Data 3/2018, 5th December.

Each participant was assigned a unique patient study number at enrolment. In trial documents, the participant’s identity is coded by study number as assigned at enrolment. The local investigator kept a subject enrolment and identification log that contains the key to the code, that is, a record of the personal identification data linked to each patient study number. This record is filed at the investigational site and can only be accessed by the investigator and the supporting site staff, and by representatives of the sponsor or a regulatory agency for the purpose of monitoring visits or audits and inspections.

### Confidentiality and data storage

The data collected were processed in accordance with the European Union legislation to ensure that requirements regarding personal data protection are met. To ensure a correct management of the data, the Joint Personal Data Management Agreement was elaborated and finally signed on 29 April 2022. Moreover, a Data Management Plan (DMP) was implemented and shared among the partners.

**Figure 4:**
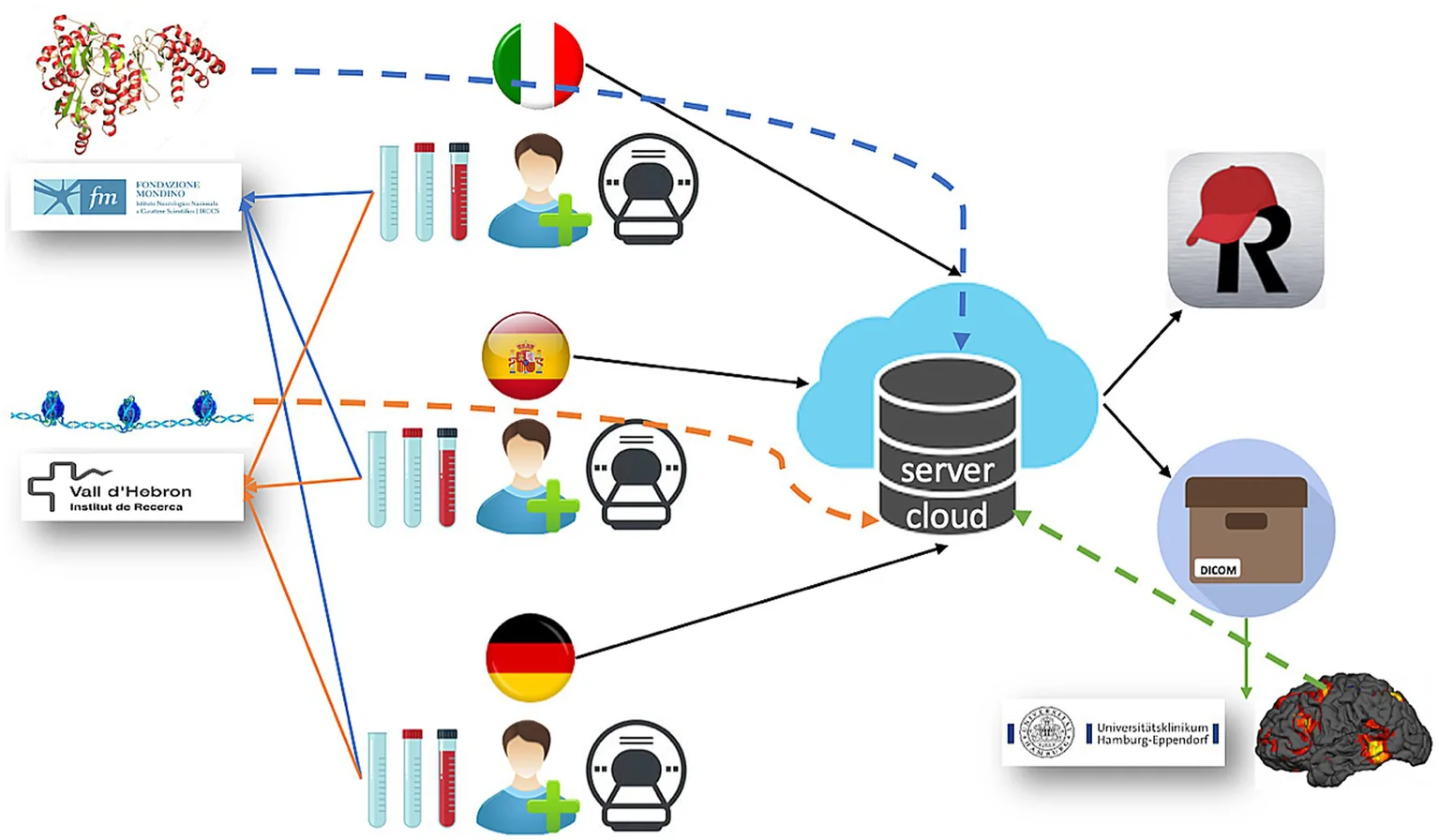
Organisation of the informatics infrastructure of the BIOMIGA Project.

All the records contained in the multimodal dataset have been anonymised or pseudonymised, when full anonymisation was not possible. They have been collected and stored in an online platform created in collaboration with a technological partner, Biomeris (Pavia, Italy) (Figure 4). All partners used the platform to acquire raw clinical, biochemical, and imaging data. Study data were collected and managed using REDCap electronic data capture tools hosted on a Server within the European Union, under the licence hosted by the IRCCS Mondino Foundation. REDCap (Research Electronic Data Capture) is a secure, web-based software platform (44) designed to support data capture for research studies, providing (1) an intuitive interface for validated data capture; (2) audit trails for tracking data manipulation and export procedures; (3) automated export procedures for seamless data downloads to common statistical packages; and (4) procedures for data integration and interoperability with external sources. The REDCap platform facilitated real-time data entry and smooth data sharing. Additionally, the creation and exchange of imaging, genetic, and psychophysical data forms enhanced collaboration and promoted methodological standardisation among consortium members, strengthening the scientific rigour of the project.

### Dissemination

The results are going to be submitted to ClinicalTrials.gov and EudraCT and actively disseminated through peer-reviewed journals, conference presentations, and social media. Moreover, a dedicated web page will be published on the website of the IRCCS Mondino Foundation, available in Italian and English for professional and general public dissemination of the protocol and of the results of this project, as soon as they are available: https://www.mondino.it.

Currently, it is possible to find the entire protocol at https://clinicaltrials.gov/study/NCT04503083.

### Data sharing

The final database using REDCap and an anonymised version is going to be made available via Zenodo^1^ after the publication of the final project’s results, upon written request.

## Discussion

We are entering a new phase in migraine prevention, characterised by the availability of mechanism-based, target-specific therapies. However, the promise of these advances may only be fully realised through a more individualised therapeutic approach in the context of precision medicine, which requires improved patient profiling and reliability of treatment response. CGRP-targeting monoclonal antibodies and gepants are transforming migraine prevention by offering the first mechanism-specific therapies with strong efficacy and good tolerability (7). However, the response to treatment varies significantly, emphasizing the need for biomarkers that can identify which patients are most likely to benefit from modulation of the CGRP pathway.

In parallel, extensive research efforts aimed at identifying biochemical, neuroimaging and genetic biomarkers of migraine have yet to yield markers that are sufficiently robust for routine clinical use in diagnosis, disease stratification or treatment selection. This disconnection between therapeutic innovation and biomarker validation highlights a critical gap in the current precision-medicine paradigm for migraine.

Within this context, the BIOMIGA project was conceived as a multimodal, biomarker-driven study focused on patients treated with erenumab, the first mAB targeting the CGRP pathway to become widely available. By integrating deep clinical phenotyping with molecular, genetic, epigenetic and advanced neuroimaging measures, BIOMIGA is expected to move beyond single-marker approaches and to identify composite biomarker signatures to support a more mechanism-informed and personalised use of CGRP-targeted preventive therapies.

### Strengths

A major strength of BIOMIGA is its prospective, multicentre design to collect extensive multimodal data in order to perform deep phenotyping of the patients. The collaboration of three specialised European headache centres, operating under harmonised protocols and shared standard operating procedures, increases sample size and enhances the external validity of the findings compared with single-centre series. The inclusion of patients living with HFEM and CM, often with several previous preventive failures, reflects real-world candidates for CGRP-targeting mABs and complements the more selected populations enrolled in pivotal randomised trials. Cross-centre quality checks and consensus procedures for resolving discrepancies further strengthen the reliability of the clinical and biomarker datasets.

Another key strength is the depth of phenotyping. BIOMIGA integrates detailed clinical and psychometric evaluations with peripheral biohumoral markers (e.g., CGRP, inflammatory cytokines, and their miRNA expression), genomic and epigenomic data (e.g., candidate SNPs and genome-wide DNA methylation), and advanced structural and functional magnetic resonance imaging. Unlike traditional databases, which handle only structured tabular data, the BIOMIGA database contains diverse data types, including text, images and specialised data such as genomic sequences. Managing the BIOMIGA dataset has presented challenges in terms of storing, processing and retrieving multiple types of data within a single, unified platform. However, we believe that this effort is necessary for understanding migraine, a complex neurosensory disorder involving interacting neuropeptide, inflammatory, vascular and network-level mechanisms. Furthermore, in the context of precision medicine, integrated biomarker panels are expected to guide therapeutic decisions, rather than single markers (15).

Focusing on a single preventive agent, subcutaneous erenumab, at the dosagike of 140 mg, is also methodologically robust. Erenumab targets the CGRP receptor, has a well-characterised pharmacokinetic profile and has demonstrated consistent efficacy and safety in both episodic and CM, therefore gaining “strong in favour” endorsement in the International Headache Society (IHS) – Società Italiana Studio Cefalee (SISC) guidelines for migraine prevention (4). Concentrating on one mechanism-specific therapy reduces pharmacological heterogeneity and facilitates the attribution of biomarker patterns to a defined biological target. The prospective collection of longitudinal data, with repeated clinical, psychometric and imaging assessments, allows evaluation of within-person changes over time and supports analyses relating biomarker dynamics to clinical response, rather than relying solely on cross-sectional comparisons. Moreover, the inclusion of a control group provides an essential reference framework, enabling the differentiation of disease-specific signals from background biological variability and thereby strengthening the validity and interpretability of the findings.

Finally, BIOMIGA adopts advanced analytical ML strategies to identify prediction models that balance interpretability and performance. This structured approach, coupled with prespecified performance metrics and internal validation, is a strength in the context of high-dimensional biomarker research.

### Limitations and future directions

The protocol was finalised in 2019, as a translational study based on the stratification of responders to CGRP-targeting mABs, when erenumab was the only mAB against the calcitonin gene-related peptide pathway widely available in the participating countries. Since then, ligand-binding mABs (fremanezumab, galcanezumab, eptinezumab) and oral CGRP antagonists (gepants such as atogepant and rimegepant) have been approved for migraine prevention, with broadly comparable population-level efficacy but distinct pharmacokinetic, pharmacodynamic and tissue-distribution profiles (2). BIOMIGA will therefore provide biomarker signatures that are directly applicable to erenumab; extrapolation for other agents targeting circulating or receptor-bound CGRP should be considered hypothesis-generating and will require independent validation.

A second limitation relates to the duration of the observation window. BIOMIGA, in line with pivotal trials, defines response at 12 weeks (45, 46). Emerging real-world evidence indicates that a minority of subjects experience “late” (beyond 3 months) responses to CGRP-targeting mABs, with clinically meaningful improvement first evident between 3 and 6 months or even later (43, 47). Contemporary clinical practice increasingly acknowledges that some patients require more than 3 months of treatment before a definitive judgement on efficacy can be made, even more than 24 months, and are therefore considered “ultra-late” responders (48). BIOMIGA may therefore have misclassified some late responders as non-responders, which could dilute biomarker associations. This should be taken into consideration when interpreting prediction models based on early response.

From a design standpoint, the open-label, non-randomised nature of the study is another important limitation. On the other hand, the stratification of patients by response to a target-specific drug represented a strong enough approach to assess response-related changes in multidisciplinary protocol. Of note, funding considerations were also important in the definition of the approach, since a placebo-controlled study would have required a larger population, a longer schedule and a more consistent funding. In addition, open-label observational designs allow inclusion of patients with high disease burden and multiple previous preventive failures, who are often under-represented in randomised controlled trials but represent a large proportion of those treated with CGRP mABs in routine practice. Future validation studies on larger populations and more stringent methodology are needed to confirm the output of this trial.

The multicentre structure, while a strength, also introduces potential heterogeneity. Despite harmonised imaging protocols and centralised molecular analysis, subtle differences in scanner hardware, software and site-specific procedures may contribute to variance in structural and functional magnetic resonance imaging metrics. Similarly, although biospecimen collection and processing were standardised and key assays centralised, residual inter-site variability in pre-analytical handling cannot be fully excluded, as well as deterioration during storage or shipment of the materials.

Specific challenges concern the measurement of circulating CGRP. CGRP has been proposed as the most promising candidate biomarker in migraine, but studies measuring its concentrations in peripheral blood and other biofluids have yielded conflicting results, owing to differences in sampling timing, assay sensitivity and specificity, and the episodic nature of peptide release (15). Recent work has started to clarify pre-analytical stability, suggesting that plasma levels are robust under controlled conditions (49), yet there is still no consensus on clinically meaningful thresholds or on the optimal sampling paradigm. BIOMIGA mitigates some of these issues through rigorous standard operating procedures and central laboratory analyses, but limited time-point measurements may not fully capture individual calcitonin gene-related peptide dynamics and could under-estimate within-person variability. Secondarily, despite evaluating patients in a pain-free state, patients living with CM determine the difficulty in having fully pain-free days within the allowed window of access, therefore possibly determining a higher variability of CGRP level or functional connectivity at the MRI scan between the ictal and interictal phases.

Other limitations are inherent to the complexity of the biomarker programme and to the application of ML approaches to high-dimensional, multimodal data. The large number of clinical, neuroimaging and molecular variables relative to the sample size increases the risk of overfitting and of identifying spurious associations, even when structured feature-selection strategies, class-imbalance handling and internal validation procedures are applied. Although BIOMIGA adopts advanced ML methodologies, including penalisation techniques and cross-validation frameworks, the resulting predictive models should therefore be regarded as exploratory and hypothesis-generating.

In addition, the absence of an independent external validation cohort limits the immediate generalisability of the proposed models. Performance estimates derived from internal validation approaches, such as out-of-bag estimation and cross-validation, may remain optimistic in the context of complex, multicentric datasets. Replication in larger, independent cohorts will be essential to confirm the robustness, stability and clinical relevance of the identified biomarker signatures.

Furthermore, while the integration of a broad range of heterogeneous features reflects the multidimensional nature of migraine pathophysiology, it may reduce model interpretability and limit direct clinical applicability. Future studies should therefore aim to derive more parsimonious and biologically informed models, hopefully, thanks to studies like this, based on a reduced set of predictors, to facilitate translation into routine clinical practice.

Finally, since the study population was exclusively recruited from tertiary headache centres in Europe during the COVID-19 pandemic, the findings may not be fully representative of patients treated in primary care settings or in non-European populations, potentially limiting generalisability. Missing data, inevitable in long and demanding protocols, may further reduce statistical power for some secondary analyses. Nevertheless, BIOMIGA provides a robust framework for the future extension and external validation of predictive models aimed at stratifying responders to CGRP-targeting mABs in larger and more diverse populations.

## Conclusion

In conclusion, the BIOMIGA trial is a phase 4 protocol funded under the ERANET-Neuron scheme. It combines data on the efficacy of a real-world, multicentre cohort of patients treated with erenumab with an unprecedented breadth of multimodal profiling, positioning it as a key step towards precision medicine in migraine. Its design strengths—harmonised procedures, deep multimodal phenotyping, healthy subjects acting as controls and advanced analytical strategies—are tempered by limitations related to historical context, open-label methodology, measurement constraints for CGRP and the challenges of high-dimensional biomarker research. Careful consideration of these factors will be essential when interpreting the forthcoming results and when designing future studies to validate and extend BIOMIGA-derived biomarker signatures across the broader spectrum of CGRP-targeted therapies.

## Footnotes

1
https://doi.org/10.5281/zenodo.15007020
